# Circulation of enterotoxigenic *Escherichia coli* (ETEC) isolates expressing CS23 from the environment to clinical settings

**DOI:** 10.1128/msystems.00141-23

**Published:** 2023-09-08

**Authors:** Carla Calderon Toledo, Astrid von Mentzer, Jorge Agramont, Kaisa Thorell, Yingshun Zhou, Miklós Szabó, Patricia Colque, Inger Kuhn, Sergio Gutiérrez-Cortez, Enrique Joffré

**Affiliations:** 1 Unidad de Microbiología Ambiental, Instituto de Biología Molecular y Biotecnología (IBMB), Carrera de Biología, Universidad Mayor de San Andrés, La Paz, Bolivia; 2 Wellcome Sanger Institute, Hinxton, Cambridge, United Kingdom; 3 Department of Microbiology and Immunology, Sahlgrenska Academy, University of Gothenburg, Gothenburg, Sweden; 4 Mathematical Sciences, Chalmers University of Technology, Gothenburg, Sweden; 5 Department of Chemistry and Molecular Biology (CMB), University of Gothenburg, Gothenburg, Sweden; 6 Department of Pathogen Biology, The public platform of the Pathogen Biology, School of Basic Medicine, Southwest Medical University, Luzhou, Sichuan, China; 7 Department of Microbiology, Tumor and Cell Biology (MTC), Karolinska Institutet, Stockholm, Sweden; University of Pretoria, Hatfield, South Africa

**Keywords:** ETEC, Bolivia, infant diarrhea, waterborne bacteria, water pollution, human-environment interphase, One Health, bacterial transmission, genomics, PhenePlate

## Abstract

Enterotoxigenic *Escherichia coli* (ETEC) is one of the leading causes of infant diarrhea in low- and middle-income countries (LMICs). Diarrheal pathogens are transmitted through environmental reservoirs; however, the bacterial clones that spread across the human-environment interface remain unexplored. We aimed to determine the relationship and clonal dissemination of ETEC between children with diarrhea and polluted water samples from a local river in La Paz, Bolivia. By using WGS and the PhenePlates phenotypic system to analyze ETEC strains, we showed that ST218 and ST410 LT+STh ETEC expressing the colonization factor (CF) CS23 were found with high frequency in both samples. The CS23 ETEC isolates were found within several STs, *E. coli* phylogroups, and across ETEC lineages. Comparative genomic evaluation and PhenePlate screening of globally distributed clinical ETEC strains suggest that the CS23 gene is likely carried on plasmids acquired independently of the bacterial chromosomal background. Clinical strains were more often multidrug-resistant (MDR) than environmental isolates and harbored the class 1 integron-integrase gene *intI1* next to the MDR cassettes. Retrospective analysis of antibiotic resistance in ETEC revealed a high frequency of MDR in clinical isolates. The LT+STh CS23 environmental ETEC isolates, showed an increased biofilm ability at environmental temperature, equal cytotoxicity, and significantly lower adherence to human epithelial cells compared to ETEC expressing other CFs. Together, we suggest that CS23 is more prevalent in ETEC than previously estimated, and the Choqueyapu River is a reservoir for LT+STh CS23 ETEC containing strains capable of causing diarrheal cases in children.

**IMPORTANCE:**

The importance of clean water cannot be overstated. It is a vital resource for maintaining health and well-being. Unfortunately, water sources contaminated with fecal discharges from animal and human origin due to a lack of wastewater management pose a significant risk to communities, as they can become a means of transmission of pathogenic bacteria like enterotoxigenic *E. coli* (ETEC). ETEC is frequently found in polluted water in countries with a high prevalence of diarrheal diseases, such as Bolivia. This study provides novel insights into the circulation of ETEC between diarrheal cases and polluted water sources in areas with high rates of diarrheal disease. These findings highlight the Choqueyapu River as a potential reservoir for emerging pathogens carrying antibiotic-resistance genes, making it a crucial area for monitoring and intervention. Furthermore, the results demonstrate the feasibility of a low-cost, high-throughput method for tracking bacterial pathogens in low- and middle-income countries, making it a valuable tool for One Health monitoring efforts.

## INTRODUCTION

Enterotoxigenic *E. coli* (ETEC) is a food- and waterborne pathogen and one of the leading causes of moderate to severe diarrhea in children, particularly in low- and middle-income countries (LMICs) and in adults traveling to endemic regions (1, 2). The Global Burden of Disease study included ETEC among the top 10 diarrheal-causing agents in the world, accounting for more than 50,000 annual deaths and 223 million cases per year (3). ETEC infection causes varying symptoms, from mild diarrhea to a severe cholera-like disease, by colonizing the small intestine using colonization factors (CFs) and the secretion of heat-labile toxin (LT) and/or heat-stable toxins (STh and STp). Over 30 different ETEC CFs have been described and shown to be associated with human infections (4 – 6), the most common being CFA/I, CS1-7, CS14, CS17, and CS21 (5). However, toxin-positive but CF-negative ETECs are frequently detected in epidemiological studies (7, 8).

Transmission of ETEC occurs via the fecal-oral route by consuming contaminated food and water (9). Studies in Bolivia have shown that not only is ETEC one of the most prevalent diarrheagenic *E. coli* pathotypes that infect children under 5 years of age (10) but also high levels of ETEC bacteria are also found in agricultural soils, farm crops, and river waters (11, 12). Since ETEC can survive for a long time in water, environmental transmission is a risk (1, 13, 14). It is unknown to what extent clinical cases have a water or food origin in La Paz, but it has been suggested that the environmental origin is highly plausible (10). La Paz, with approximately 1 million inhabitants, does not have a wastewater treatment plant, and wastewater is discharged directly into the Choqueyapu River. Downstream of the Choqueyapu River, many communities use river water for crop irrigation, whose products are mainly commercialized in markets in the city of La Paz (11, 12). In a recent global study of water pollution, some rivers in the La Paz Basin were reported to be among the most polluted rivers, with high levels of pharmaceutical residues (15). These reports highlight the need to carry out One Health strategy studies that help explain the largely unknown interactions between humans and the environment in the emergence and transmission of diarrheal diseases.

In the present study, we characterize ETEC isolates from diarrheal cases of hospitalized children and from the water samples collected on the Choqueyapu River in La Paz, Bolivia. Whole-genome sequencing (WGS) was used to determine the relationship and clonal dissemination of ETEC between clinical and environmental samples. We also compared the genomic results of ETEC diversity and the relationship of bacterial strains from different sources using a low-cost phenotypic fingerprinting system (PhenePlate or PhP method). We found that a surprisingly high number of ETEC isolates harbored a less common CF, CS23, which is usually undetected due to the lack of established detection methods for this CF. Therefore, CS23-positive isolates are grouped into CF-negative ETEC in most studies but may be more prevalent in the environment and clinical settings than previously anticipated.

## RESULTS

### Whole-genome analysis of clinical and environmental strains reveals that CS23 circulates in both settings

Clinical and environmental isolates were collected in 2013–2014 and 2014, respectively, to determine the spread of ETEC between the two interfaces and characterize the ETEC isolates using WGS (Table 1; Table S1). In total, 30 ETEC strains with positive PCR results for ETEC toxins were included in the study, of which 11 isolates were collected from hospitalized children aged 5–60 months with acute diarrheal disease. During the same sampling period, 19 isolates were obtained by filtering shallow water at six different sites along the Choqueyapu River, where its waters are used for agricultural irrigation (Table S1). All isolates positive for toxins in PCR were whole-genome sequenced, and genes and gene clusters encoding toxins (LT and/or STh or STp) and colonization factors (CFs) were identified and extracted using an open-source database (https://github.com/avonm/ETEC_vir_db). Eleven combinations of toxins/CFs were identified; clinical isolates had a greater diversity of virulence factors that accounted for eight virulence profiles, while environmental strains displayed four different profiles (Table 1). We found that LT+STh CS23 was the most prevalent virulence profile among environmental isolates (15/19), and the same profile was found in three (3/11) clinical isolates. Four isolates were found to harbor a cluster of genes that encode a putative chaperone-usher assembled pili, where three of the isolates corresponded to clinical samples. The gene cluster encoding CS23 in two isolates (MALC (1) and ET-26) was located on large contigs 89,895 bases and 82,343 bases, respectively. This enables us to investigate the context of CS23 gene clusters. Both contigs contain plasmid-specific genes, and they were precited as IncI1-I (alpha)-replicons. BLASTn analysis comparing the two contigs shows that they are near identical with a 97% coverage and 100% sequence identity. The contigs also harbor multiple conjugal genes indicating that the plasmids harboring CS23 in these two isolates may be mobile. The CS23 gene cluster of the other genomes was located on smaller contigs with short flanking regions limiting context analysis. Comparison of the CS23 major subunit (AalE) extracted from the isolates with the CS23 variant revealed five major AalE types (AalE-1–5) (Fig. S1; Table S1). The CS23 variants AalE-2 (ET13, ET18, and M5KL (1)) and AalE-5 (ET11, ET15, ET21, ET22, ET26, and 149 B7 (1)) were found among clinical and environmental samples. The other CS23 variants, AalE-1 and AalE-4, were present exclusively in a few environmental isolates, while AalE-3 was found in the clinical isolate MALC (1) (Fig. S1; Table S1). Regarding the other clinical isolates, they had virulence profiles commonly reported in the literature (CS2+CS3+CS21, CS1+CS3+CS21, CS6, and CS21-only) (16, 17). Additional characterization of non-classical ETEC virulence factors identified two serine proteases encoded by *eatA* and the two-partner apparatus *etpBAC* of the EtpA fimbria (Table 1).

**TABLE 1:** Phenotypic and genomic characteristics of ETEC isolates collected in Bolivia

Isolate ID	Source	Type	Toxin profile	CF profile	Non-classical VF^*f*^	MLST (ST)	Phylo-group	ETEC lineages
ET5	Water	River	LT^*b*^ ^*,^c^*^+STh	CS23^*c*^		69	D	ND
ET6	Water	River	LT	Putative CU-pili		155	B1	19
ET7	Water	River	LT+STh	CS23		58	B1	19
ET8	Water	River	LT+STh	CS23		949	B1	19
ET9	Water	River	LT+STh	CS23		410	C	16
ET10	Water	River	LT+STh	CS23		58	B1	19
ET11	Water	River	LT+STh	CS23		218	A	12
ET12	Water	River	LT+STh	CS23^*c*^		949	B1	12
ET13	Water	River	LT+STh	CS23		410	C	16
ET14	Water	River	STh	CS23		10	A	15
ET15	Water	River	LT+STh	CS23		949	B1	19
ET17	Water	River	LT+STh	CS23^*c*^		10	A	15
ET18	Water	River	LT+STh	CS23		410	C	16
ET19	Water	River	LT+STh	CS23		10	A	15
ET21	Water	River	LT+STh	CS23		218	A	12
ET22	Water	River	LT+STh	CS23		949	B1	19
ET23	Water	River	LT	CS6+CS8^*d*^	*eatA*	3,931	A	4
ET25	Water	River	LT	CS6+CS8^*d*^	*eatA*	3,931	A	4
ET26	Water	River	LT+STh	CS23		949	B1	19
51 LAE	Clinical	AD^*a*^	LT	Putative CU-pili	*etpBC^g^*	10	A	15
78 CRE	Clinical	AD^*a*^	LT+STh	CS2+CS3^*e*^+CS21^*d*^	*eatA etpBC^g^*	4	A	2
149 B7	Clinical	AD^*a*^	LT+STh	CS23		218	A	12
169 LAF	Clinical	AD^*a*^	LT	CS6		10	A	15
250 B7 (1)	Clinical	AD^*a*^	LT	Putative CU-pili		155	B1	19
284 B7 (1)	Clinical	AD^*a*^	STh	CS21		48	A	4
570 LAE	Clinical	AD^*a*^	LT	CS1+CS3+CS21		4	A	1
619 B8 (1)	Clinical	AD^*a*^	STh	CS21		48	A	4
724 B9 (1)	Clinical	AD^*a*^	LT	CS13+putative CU-pili	*eatA etpBC^g^*	46	A	22
M5 KL (1)	Clinical	AD^*a*^	LT+STh	CS23		410	C	16
MALC (1)	Clinical	AD^*a*^	LT+STh	CS23		Novel	A	15

^*a*^ AD: acute diarrhea.

^*b*^ LT gene duplicated.

^*c*^ Interrupted operon (could be due to sequencing issues).

^*d*^ *cfaD* gene present.

^*e*^ Missing *cstB* gene from the CS3 operon.

^*f*^ VF: virulence factors.

^*g*^ Disrupted contigs.

### Circulation of CS23 ETEC isolates with the same ST group between clinical and environmental settings

To assess the heterogenicity of ETEC strains and their genetic relationships, we performed *in silico* multilocus sequence typing (MLST) and *E. coli* phylogroup typing (Table 1). The MLST results showed that our 30 ETEC strains belonged to 11 different sequence types (STs), with three exclusives to clinical isolates (ST4, ST48, and ST46) and four to environmental isolates (ST58, ST69, ST949, and ST3931). Furthermore, the presumed high-risk clones of *E. coli* ST410 and ST218, ST155, and ST10 were found in both clinical and environmental samples. Based on virulence profiles, LT+STh CS23 strains were not associated with a specific ST type or clonal group.

**Fig 1:**
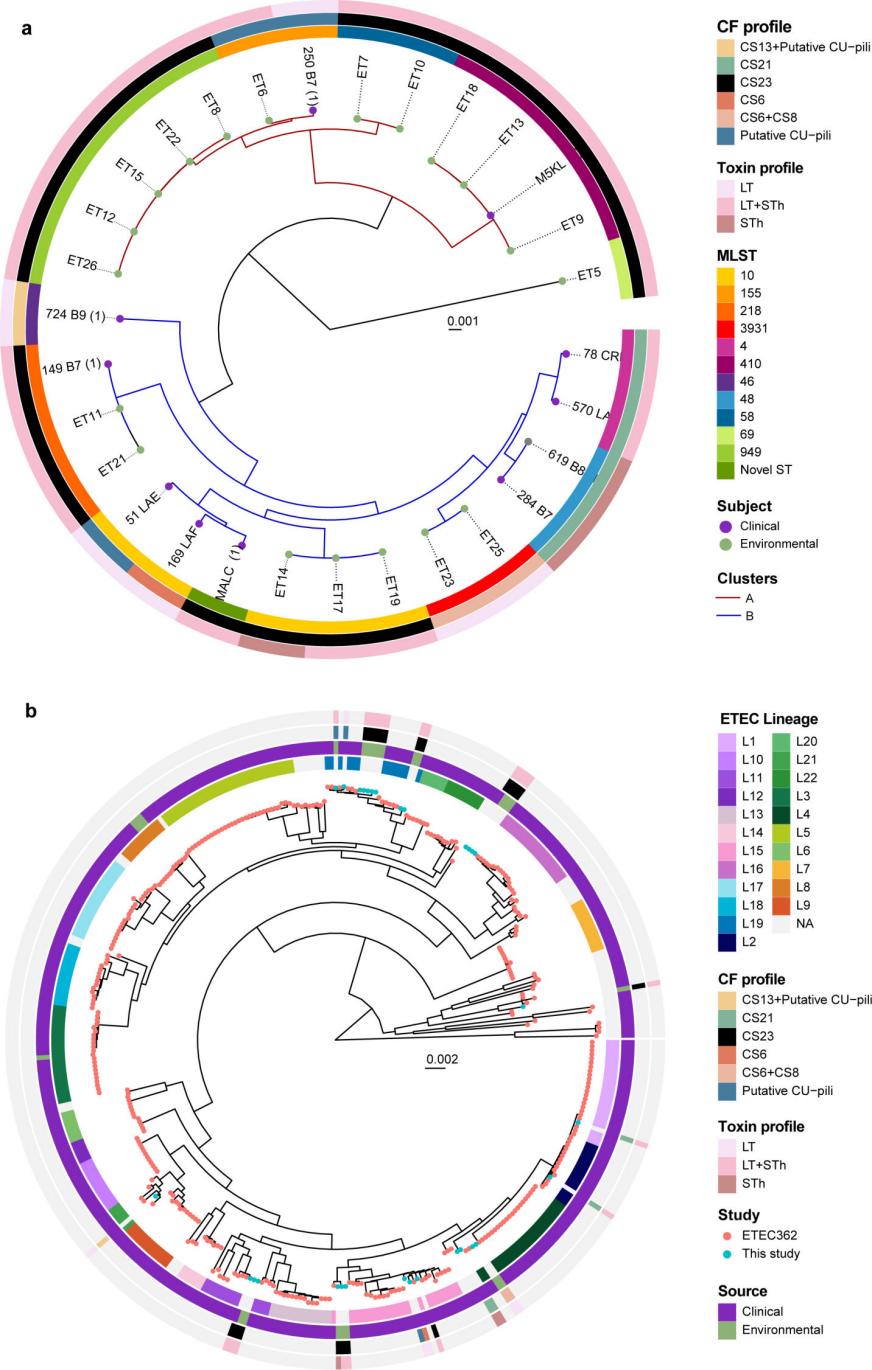
Whole-genome phylogenetic analysis of ETEC. Maximum likelihood midpoint-rooted phylogenetic trees based on single-nucleotide polymorphism (SNP) differences in (**a**) 30 ETEC genomes collected in Bolivia and (**b**) in context with 362 selected ETEC genomes with representative virulence profiles isolated from indigenous patients and travelers between 1980 and 2011 from endemic countries (Asia, Africa, and North, Central and South America). The tip of the branches is color-coded according to the sample’s origin (clinical/environmental in this study and the 362 reference ETEC genomes). The colored rings represent the respective MLST and CF profiles, and the lineages L1–L21 are indicated. The scale bar represents the substitutions per variable site.

The analysis of 30 ETEC strains exhibited a high level of diversity, as they were distributed among four *E. coli* phylogroups (A, B1, C, and D) (Table 1). Phylogroup A was primarily linked to clinical isolates expressing CS1–CS3, CS6, CS13, or CS21 as we have shown previously (18), while phylogroups A, B1, and C were represented among environmental isolates. Phylogroup C included clinical and environmental strains, and all of which were ST410. CS23-positive ETECs were not confined to any specific phylogroup. Phylogenetic analysis of the 30 ETEC isolates showed a consistent clustering pattern with MLST groups and demonstrated specific clusters of environmental and clinical ETEC with a high degree of genetic relatedness (Fig. 1a). The phylogenetic analysis of ETEC genomes revealed two clusters, A and B. Cluster A contained 11 environmental and 2 clinical genomes, including subgroups ST949, ST155, ST58, and ST410. The ST155 and ST410 groups had both environmental and diarrheal isolates. Cluster B comprised 9 clinical and 7 environmental isolates with ST218 subgroup including mixed-sources isolates. ST10 isolates were found in both clinical and environmental samples but not grouped together. Environmental ST3931 isolates were closely related to clinical ST4 and ST48 isolates (Fig. 1b). Overall, our data confirmed the presence of two LT+STh CS23 ETEC clones (ST410 and ST218) in the Choqueyapu River, which cause diarrheal cases during the time of sampling and observed high genetic diversity among clinical isolates and close relatedness between clinical and environmental isolates.

To identify whether Bolivian strains belonged to known ETEC lineages, we built a phylogenetic tree incorporating reference genomes of 362 clinical ETEC strains collected from children and adults with diarrheal disease in endemic countries (Asia, Africa, and Central and South America) over a 30-year period (1980–2011) (18) (Fig. 1b). Our results revealed that the new Bolivian ETEC strains belonged to eight ETEC lineages (L1, L2, L4, L12, L15, L16, L19, and L22) (18). Surprisingly, all environmental isolates were genetically close to clinical ETEC strains distributed worldwide. Only six Bolivian strains in this study (570_LAE, 78_CRE, 284_B7, 619_B8, ET23, and ET25) belonged to the major ETEC lineages (L1–L5), which include the most prevalent virulence profiles. Their virulence profiles were consistent with the virulence profile associated with each lineage in the von Mentzer study (18). For example, L4, which commonly encompasses ETEC strains expressing CS6, CS6+CS8, and CS21, also clustered with the STh CS21 and LT CS6+CS8 ETEC isolates from this study. Interestingly, ETEC strains expressing CS23, CS6, CS13, and CF-negative fell into ETEC lineages (L12, L15, L16, L18, and L22), where most strains do not express any of the known ETEC CFs. The prevalence of CF-negative ETECs is partly due to a lack of standardized detection methods for several CFs, such as CS23, but might also suggest the presence of undiscovered CFs. Similarly to the MLST results, LT + STh CS23 genomes were distributed across four ETEC lineages (L12, L15, L16, and L19). A representative ETEC LT+STh CS23 isolated from a diarrheal case from the von Mentzer reference collection was found in L11. These results demonstrated a close relationship between environmental and clinical isolates associated with acute diarrhea cases globally. Moreover, we showed that LT+STh CS23 ETEC isolates were present in highly diverse genetic backgrounds, and we suggest that CS23 genes might be harbored in plasmids mobilized by horizontal gene transfer. Given the widespread presence of CS23 ETEC in this study, this CF should be considered in future studies of ETEC diarrhea.

### Antimicrobial susceptibility profiles, resistance genes, and incompatibility groups

**Fig 2:**
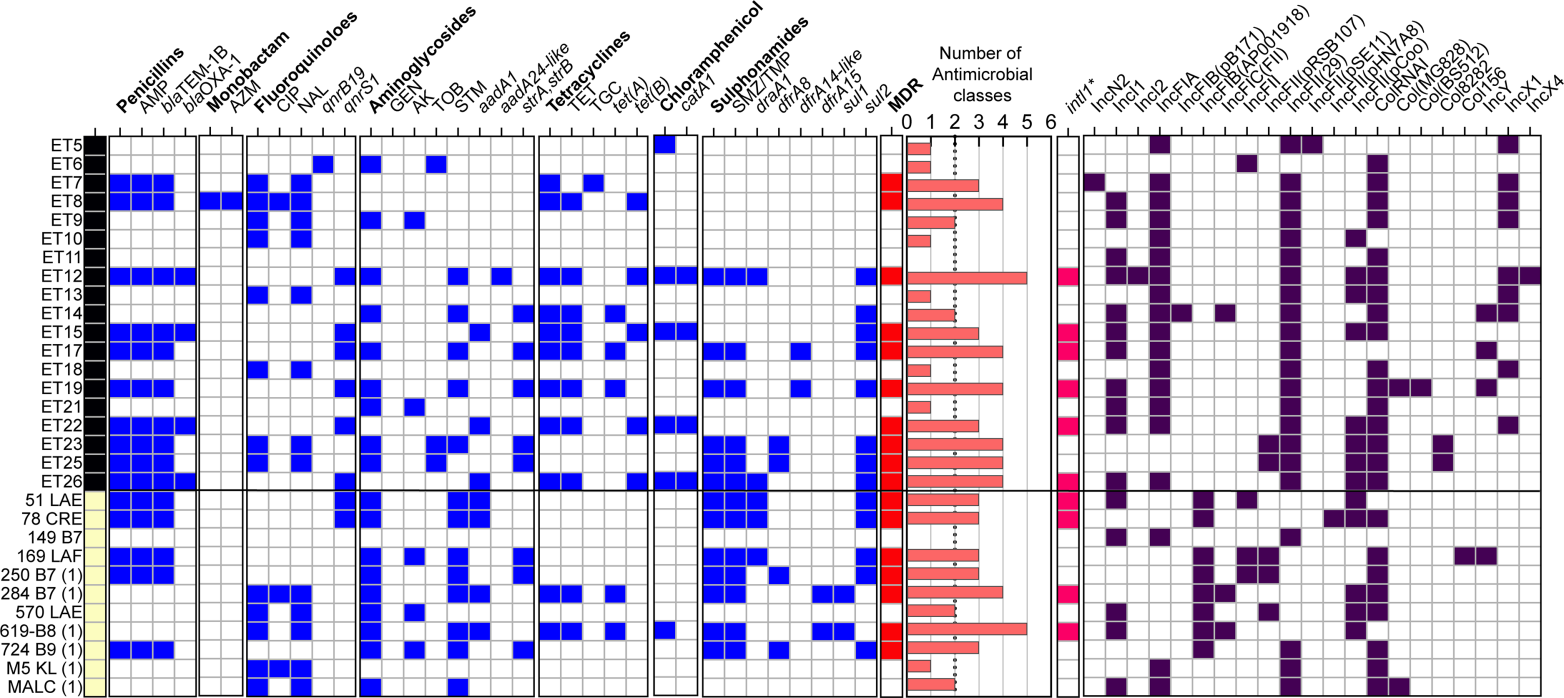
Phenotypic and genomic characterization of antibiotic resistance among ETEC isolates. Environmental (black) and clinical (yellow) samples were subjected to a phenotypic disk diffusion test (capital letters). Antimicrobial resistance genes were extracted from ETEC genomes (in italics). The blue boxes represent resistant/positive isolates, and the red boxes indicate multidrug resistance (≥3 classes of antibiotics). The histogram illustrates the number of antimicrobial classes (written in bold) in which the ETEC strains were phenotypically resistant. Genomic identification (≥90% nucleotide identity and ≥90% nucleotide identity) of the mobile element class 1 integron (*intI1*) (GenBank: AEH26333.1). Characterization of different Incompatibility Group (Inc) plasmids detected in bacterial genomes. AMP, ampicillin; AZM, azithromycin; CIP, ciprofloxacin; NAL, nalidixic acid; GEN, gentamycin, AK amikacin; TOB, tobramycin; STM, streptomycin; TET, tetracycline; TGC, tigecycline; and TMP/SMZ, trimethoprim-sulfamethoxazole.

The antimicrobial susceptibility tests (ASTs) of the 30 ETEC isolates against 16 antimicrobials from nine antibiotics classes revealed phenotypic resistance for 11 of the 16 tested antimicrobials (Fig. 2). 90% of clinical and 95% of environmental isolates showed resistance to at least one drug class with no resistance to β-lactams (cefotaxime, cefpodoxime, meropenem, imipenem) or gentamicin. MDR (resistance to three or more classes of antibiotics) was more common in clinical isolates (63%) than in environmental isolates (52%). Streptomycin (STM) resistance was significantly higher in clinical isolates compared to environmental isolates (73% versus 26%, Fisher’s *P*-value = 0.0119) (Table S2). Trimethoprim-sulfamethoxazole (TMP-SMX) resistance was detected twice more frequently in clinical isolates (64%) than in environmental isolates (32%). Ampicillin resistance was also common, with environmental isolates showing a frequency of 52% and clinical isolates at 45%. While resistance to ciprofloxacin—a widely used antibiotic for the treatment of acute diarrhea in Bolivia (19)—was low in both groups, nalidixic acid resistance was observed in 43% of the isolates. According to the ST clonal groups, ST48 (*μ* = 4.5), ST3931 (*μ* = 4), ST949 (*μ* = 3.8), and ST10 (*μ* = 3.2) displayed the highest average (*μ*) of phenotypic antibiotic resistance per strain. Interestingly, the high-risk MDR clone of *E. coli* ST410 included non-MDR isolates with resistance to nalidixic acid and, occasionally, to ciprofloxacin or amikacin. ST218 isolates were sensitive to all antibiotics (Fig. 2).

Genomic screening of the 30 ETEC genomes identified 17 antimicrobial resistance genes (ARGs) from six different classes of antibiotics (Fig. 2). Environmental and clinical isolates shared eight ARGs that confer resistance to aminoglycosides (*aadA1* and *strA*/*strB*), sulfonamides and trimethoprim (*sul2*, *drfA1,* and *drfA8*), penicillin (*bla*_TEM-1b_), fluoroquinolones (*qnrS1*), and tetracyclines (*tetA*). No statistical differences in ARG prevalence were found between the two groups, with similar average number of ARGs (2.9 versus 2.8). Environmental strains had higher ARG diversity and contained exclusive ARG alleles such as *aadA24*, *dfrA14-like*, *bla*_OXA-1_, *qnrB19, tetB*, and *catA1*, while *sul1* and *dfrA15* alleles were only found in clinical isolates. No carbapenemases, rifampicin, macrolides, or colistin resistance genes were detected. ST218 (ET11, ET21, and 149 B7) and ST410 (ET9, ET13, ET18, and M5 KL) did not carry any ARG, but some of them exhibited phenotypic resistance to fluoroquinolones, possibly due to the acquisition of a mutation in DNA gyrase and topoisomerase IV and not plasmid-borne resistance genes. As shown in Fig. 2, the comparison of the AMR phenotype and genotype correlated to a large extent, particularly in strains resistant to ampicillin (*bla*_TEM-1B_ *+ bla*_OXA-1_), tetracycline (*tetA+tetB*), TMP-SMZ (*draA1*, *draA8, dfrA15*, *sul1,* and/or *sul2*) and chloramphenicol (*catA1*). However, exceptions were observed in ET5 and 619-B8 isolates, which tested negative for any chloramphenicol-resistant gene.

Further analysis of the genomic context of ARGs in MDR clonal groups revealed the presence of class 1 integron integrase (*intI1*) in proximity to ARGs (Fig. 2; Fig. S2). However, ET12, ET26, and 51 LAE, which were positive for *intI1*, had contigs that were too small for analysis. The *intI1* gene, found in a variety of antibiotic-resistant bacteria in both environment and humans sources, serves as an indicator of horizontal gene transfer, which occurs naturally or results from human activities such as antibiotic use and fecal pollution (20). The types of MDR cassettes were identified adjacent to *intI1* in clinical and environmental strains. The first gene arrangement, consisting of *sul1-qacEdelta1-addA1* (or 15)*-dfraA1* (or 15)*-intI1*, was exclusively identified in clinical isolates. The second arrangement formed by *addA1-bla*_oxa-1_-intl1 with *catA1* gene downstream was detected in environmental genomes ET15 and ET22. The ET17 and ET19 contigs, which share >98% identity, contained the *dfraA14* allele adjacent to the *intI1* gene (Fig. S2). A BLASTn analysis of these contigs revealed that most, though not all, had high coverage and showed significant similarity plasmids found in ETEC and other *E. coli* and *Enterobacterales* (*Shigella dysenteriae* and *Klebsiella pneumoniae*). For instance, contigs in 284 B7 (1) and 619 B8 (1) showed high similarity with L4_E1441_ ETEC plasmid 2 (pAvM_E1771_17 LR883013.1), characterized by carrying *sul1*, *tetA* genes, and the *Ing* operon encoding CF CS21 (21). Notably, the ET22 MDR cassette was integrated into a 93,402-nucleotide long chromosomal contig (Fig. S2).

Our study investigated plasmid replicon types in ETEC isolates. We identified a diverse range of incompatibility groups, including IncF (IncFIA, IncFIB, IncFIC, and IncFII), IncN (IncN2), IncI (IncI1 and IncI2), Col-like (ColRNAI), and IncY and IncX (IncX1 and IncX4). IncF was present in all ETEC strains, while the distribution of IncI, Col-like, and IncY was similar between clinical and environmental isolates. IncX (ET5, ET7-9, ET12-14, ET18, and ET22) and IncN2 (ET7) plasmids were only found in environmental strains. No specific association between Inc plasmid groups and ARGs was observed, but all LT+STh CS23-positive isolates harbored IncFIA+IncFII (*n* = 29). Various pMLST types were detected within the IncF plasmid (B18, B53, B45, B52, and A16:B45) with B18 variants common in ST3931 LT CS6+CS8 isolates and B53 found in 250 B7(1) clinical strains. Other IncF plasmids had unknown STs.

### ETEC isolates expressing CS23 showed elevated biofilm formation at 20°C

Biofilms are multicellular aggregates formed on surfaces, used as a bacterial strategy to enhance persistence in aquatic environments and the host’s gastrointestinal tract (22). We tested the colony morphotypes of ETEC isolates at temperatures mimicking environmental conditions (20°C) and the mammalian gastrointestinal tract (37°C). The Congo Red assay results showed that 20°C induced *rdar* morphotype expression (red, dry, and rough phenotype, expressing curli fimbriae and cellulose and indicative of elevated biofilm formation) in most ETEC isolates (14 environmental isolates and 6 clinical isolates), while 37°C commonly induced *bdar* phenotype (brown, dry, and rough with curli expression) in both clinical and environmental isolates (Table 2; Fig. S3). A higher frequency of saw morphotype (smooth and white expressing neither curli nor cellulose) was observed at 20°C than at 37°C. No association was found between the MLST groups and biofilm ability, although strains belonging to ST58, ST410, ST10, ST155, and ST949 showed higher biofilm production compared to ST218 and ST3931 strains. Among the 20 *rdar*-positive isolates, 15 were found to be CS23 positive.

**TABLE 2:** Effect of temperature on the biofilm formation of ETEC isolates^*a*^

Morphotype change	Environmental		Clinical	
20°C→37°C	n (%)	ST group (isolate ID)	n (%)	ST group (isolate ID)
*rdar→rdar*	5 (26.3)	ST69 (ET5), ST410 (ET9), ST10 (ET14, ET17, ET19)	3 (27.3)	ST155 (250 B7 (1)), ST218 (149 B7), ST410 (M5 KL (1))
*rdar→bdar*	6 (31.6)	ST155 (ET6), ST58 (ET7), ST949 (ET8, ET15, ET12), ST410 (ET13)	2 (18.2)	ST10 (169 LAF), ST48 (619-B8 (1))
*rdar→pdar*	0 (0.0)		1 (9.1)	ST48 (284 B7 (1))
*rdar→saw*	3 (15.8)	ST58 (ET10), ST949 (ET22, ET26)	0 (0.0)	
*bdar→rdar*	1 (5.3)	ST410 (E T18)	0 (0.0)	
*bdar→pdar*	0 (0.0)		1 (9.1)	Novel ST (MALC (1))
*bdar→saw*	0 (0.0)		1 (9.1)	ST10 (51 LAE)
*bdar→bdar*	0 (0.0)		2 (18.2)	ST4 (570 LAE), ST46 (724 B9 (1))
*saw→saw*	3 (15.8)	ST218 (ET21), ST3931 (ET23, ET25)	1 (9.1)	ST4 (78 CRE)
*saw→bdar*	1 (5.3)	ST218 (ET11)	0 (0.0)	

^*a*^ Morphotype distribution in environmental and clinical isolates of ETEC. Morphotype: *rdar* (cellulose/curli fimbriae), *pdar* (cellulose/no fimbriae), *bdar* (no cellulose/curli fimbriae), and *saw* (no cellulose/no curli fimbriae). The ETEC strains were grown at 20°C or 37°C.

### Clinical isolates exhibit higher adherence to mammalian cells but similar cytotoxicity levels as environmental isolates

**Fig 3:**
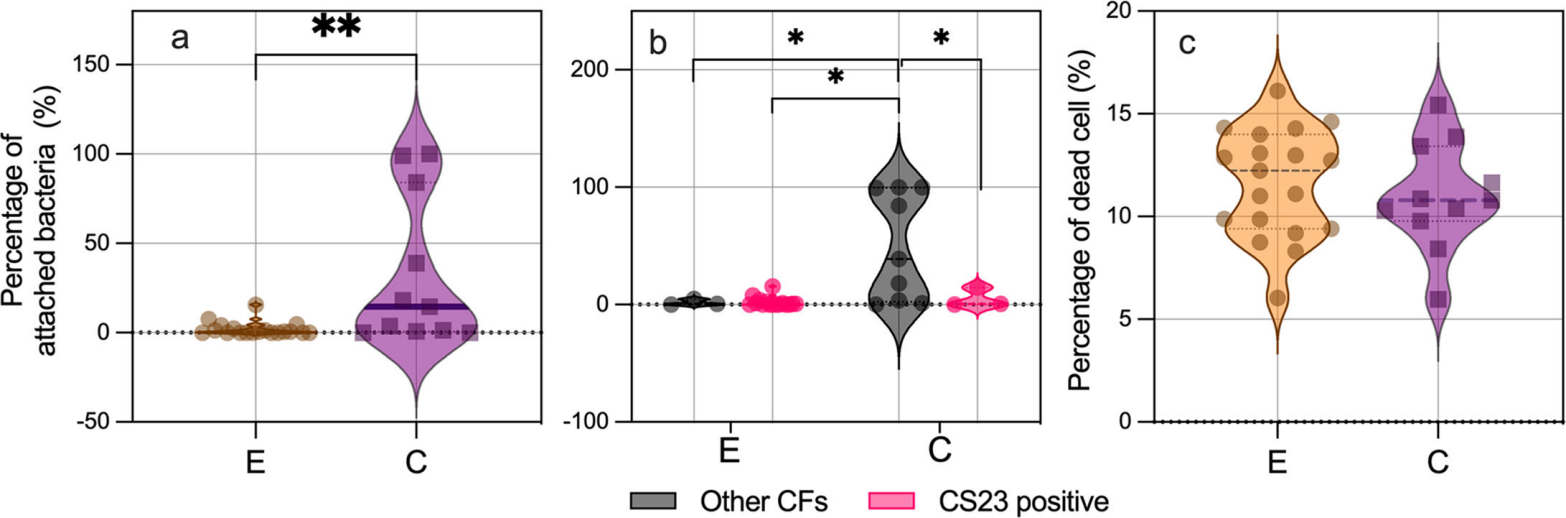
Bacterial adherence and cytotoxicity to human epithelial cells. (**a**)Differences in the number of bacteria attached to Caco-2 monolayers according to the source of isolation (environmental and clinical) or (**b**)ETEC expressing CS23 versus ETEC isolates expressing other CFs (non-CS23) of each group. C, clinical and E, environmental. Assays were performed in triplicate, and the violin plots show the distribution of the data and the mean and SD. The asterisks indicate a significant difference in the unpaired *t*-test (*P < 0.05 and **P < 0.01) using GraphPad Prism 8.00. (**c**)Cytotoxicity to Caco-2 cells infected by environmental and clinical ETEC isolates.

ETEC adhesion was evaluated in the Caco-2 intestinal epithelial human cell line, revealing that clinical ETEC isolates had a significantly higher average adhesion rate of 38.5% compared to 2.1% for environmental isolates (*P* < 0.01) (Fig. 3a). Further analysis comparing the binding ability of CS23-expressing isolates with non-CS23-expressing isolates (i.e., CS21, CS6, CS6+CS8, CS13, CS1, CS2, CS3, and putative CU-pili) revealed that non-CS23-expressing isolates had significantly higher binding ability compared to any other group (*P* < 0.05, Fig. 3b). A large majority (11/19) of ETEC isolates expressing CS23 from both groups were unable to attach to Caco-2 cells (<1% of attached bacteria). A lack of adherence was also observed in a few isolates with the following CF profile: CS21 (284 B7 (1)), and CS6+CS8 (ET23 and ET25). These results indicate that CS23 alone is not sufficient to mediate bacterial adherence to host epithelial cells, highlighting the importance of other colonization factors.

ETEC infection can disrupt/destroy the epithelial barrier, increase permeability, and induce an inflammatory response that damages intestinal cells (23). Bacterial cytotoxicity in Caco-2 cells was assessed by the presence of dead/live cells identified by fluorescence microscopy. The results showed that even though clinical isolates had a higher binding phenotype than environmental strains, both groups of strains were equally cytotoxic to Caco-2 cells, as shown in Fig. 3c.

### Circulation of ETEC isolates between the clinic and the environment is corroborated by PhenePlate (PhP) typing

**Fig 4:**
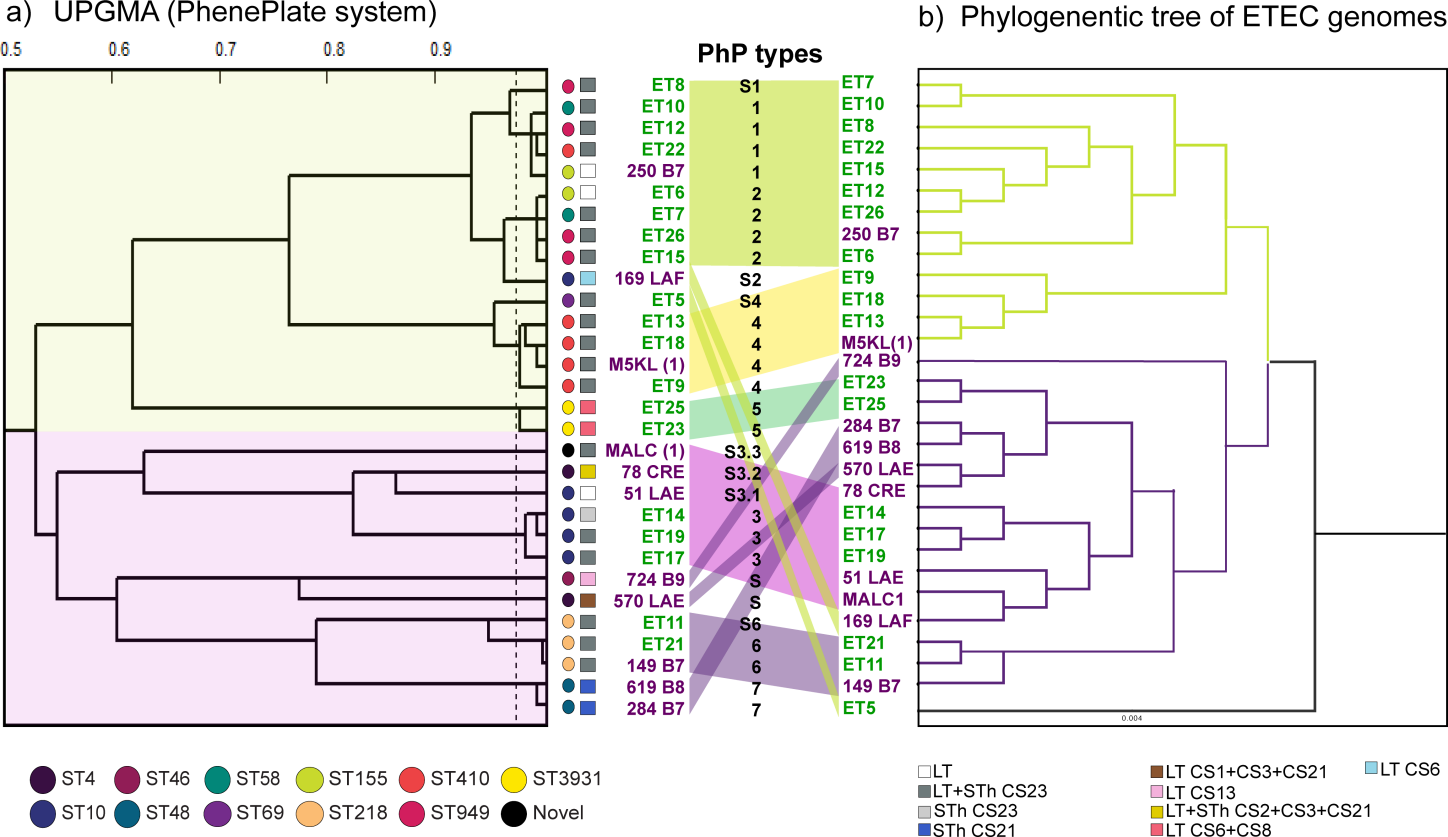
UPGMA dendrogram derived from clustering of the PhP types compared with the genome-based phylogenetic tree of the 30 ETEC genomes. (**a**)UPGMA dendrogram derived from clustering PhP typing data from clinical and environmental ETEC isolates. The dotted lines indicate the levels of identity (correlation coefficient > 0.975). A colored link joins the same strains between two clustering methods. The major clusters were colored. (**b**)Lineal tree modified from Fig. 1a.

The affordability of DNA sequencing costs has improved, but the high cost of equipment, reagents, and limited resources in LMICs still present barriers to researchers accessing genomic technologies. Therefore, it is essential to develop complementary, low-cost methods for detecting bacterial isolates. Here, we used PhenePlate (PhP) typing to assess the bacterial relatedness of clinical and environmental isolates and compare the results to those from WGS. The PhP method is a simple and rapid *E. coli* subtyping method that compares the carbon source metabolism patterns of each isolate using the UPGMA (Unweighted Pair Group Method with Arithmetic Mean) clustering method to describe the clonal relationship between bacterial isolates. Isolates with fingerprint similarities greater than 0.975 are considered identical and assigned to the same PhP type. As shown in Fig. 4a (Table S3), 30 ETEC isolates subjected to PhP typing yielded seven PhP types and seven single PhP types, which represent isolates with a unique fingerprint (*S*n).

When comparing the UPGMA dendrogram and the phylogenetic analysis, both trees displayed congruent tree topologies and similar clustering pattern. For example, ST410 and ST218 genomes were clustered together and assigned distinctive PhP types such as P4 and P6, respectively, corroborating the findings of circulation of genotypically and phenotypically highly similar isolates across clinical and environmental settings. Another example is the P1 and P2 isolates which clustered together in the UPGMA tree (correlation coefficient > 0.9) and included the genetically closely related ST58, ST155, and ST949 from cluster A as shown inFig. 1a. Additional correlations between PhP types and ST groups were also observed in P3, P5, and P7 that corresponded to ST10, ST3931, and ST48 isolates, respectively. Isolates with a single PhP type showed the most significant variation between the two methods. Overall, the PhP screening method demonstrated consistency with the genomic resolution provided by the MLST technique, highlighting that the PhP method is a powerful tool for detecting differences in circulating pathogenic strains from various sources.

### Retrospective PhenePlate profiling of ETEC clinical isolates from endemic countries

Next, we assessed the clonal relationship of our strains in the context of an ETEC collection of isolates from endemic countries, including Bolivia, by the PhP method. The bacterial collection comprised 152 ETEC strains from children under 5 years of age with acute diarrhea, collected between 1980 and 2011. Among these, 106 were Bolivian strains from previous studies (10, 24) and the rest were from South (Argentina), Central (Guatemala) and North America (Mexico), Asia (Bangladesh and Indonesia), and Africa (Morocco and Egypt) (18, 25 – 27) (Table S4). In addition, reference ETEC strains of the main lineages were included in this collection (E925: L1, CS1+CS3+CS21; E1649: L2, CS2+CS3+CS21; E1441; L4, CS6+CS21; E1779: L5, CS5+CS6; and E562: L6 CFA/I+CS21 and L7: E1373 CS6) (21).

**Fig 5:**
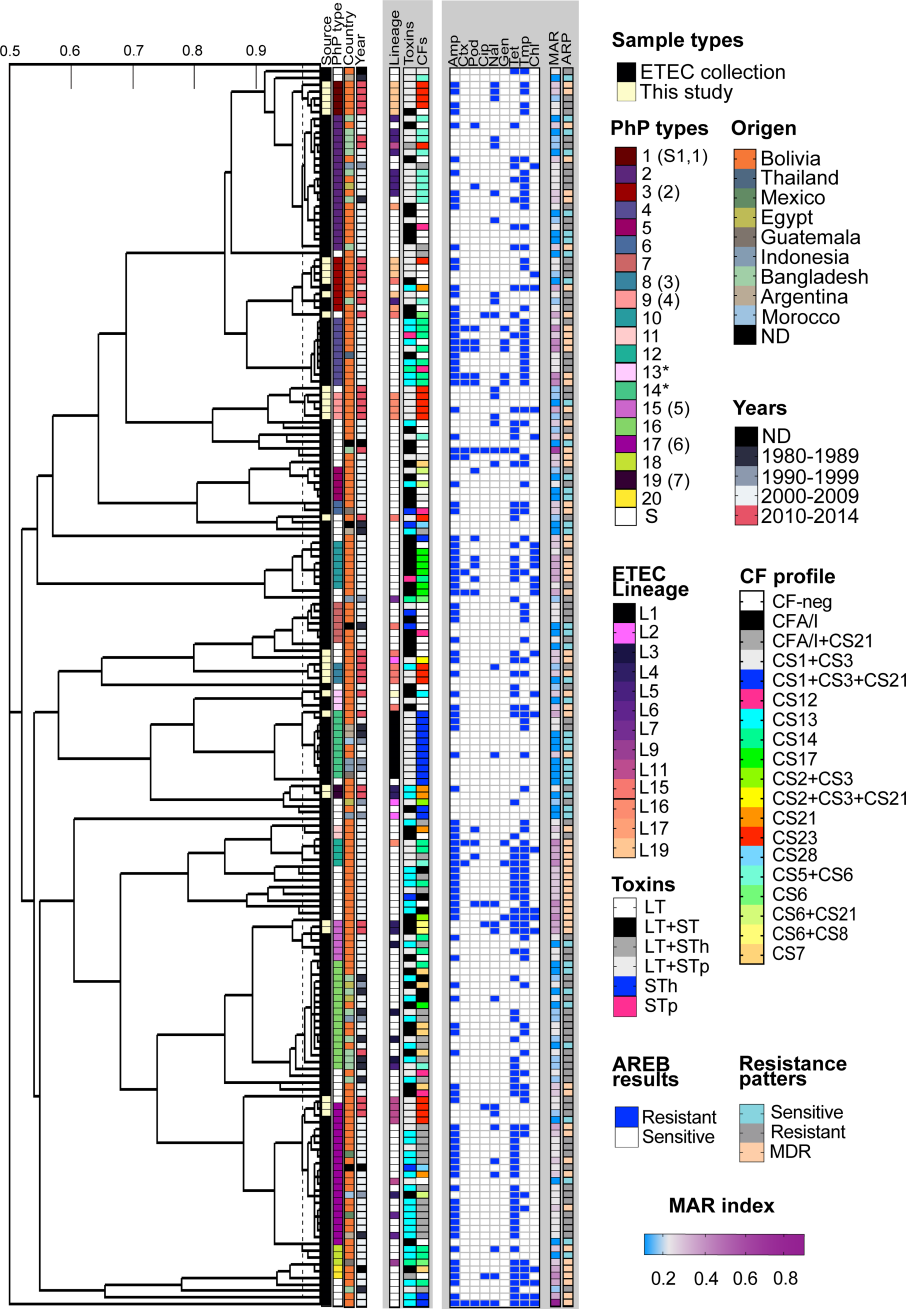
Comparison of phenotypic fingerprints from a collection of clinical Bolivian ETEC strains and isolates obtained in this study. The dendrogram shows the clustered PhP typing data, including the antibiotic resistance patterns obtained parallel by the AREB system. Information about the place and year of isolation, ETEC lineage, toxin, and CF profile are indicated in the legends in the figure. ETEC lineage information was included only for isolates from von Mentzer’s ETEC collection. Isolates from this study yielded different PhP numbers and the former PhP assignment from Fig. 4 is shown in parentheses. Antibiotic resistance to the nine AREB antibiotics, the multiple antibiotic resistance index (MAR), and antibiotic resistance patterns such as sensitivity, resistance, and multidrug resistance (MDR, ≤3 classes of antibiotics) were incorporated into the heatmap. The white coloration in the MAR index gradient indicates the critical limit of the MAR of 2. AMP, ampicillin; CTX, cefotaxime; POD, cefpodoxime; CIP, ciprofloxacin; NAL, nalidixic acid; GEN, gentamycin; TET, tetracycline; and CHL, chloramphenicol.

Of the 182 ETEC tested, 20 PhP types were identified (Fig. 5; Table S5). Most of the clinical and environmental isolates of this study were spread across the PhP dendrogram, forming their own clusters. For example, environmental CS23 ETEC strains P3 (ET6, ET7, ET26, and ET15), previously assigned as P2 in Fig. 4, clustered with clinical Bolivian (CS21 E2381 and CF-neg E2404) and Bangladeshi (CS5+CS6 E5082) strains from the ETEC collection. Additionally, the 724 B9 and 570 LAE, formerly described as having unique PhP types, were clustered with P13 and P14 ETEC isolates, respectively. The P13 group included two more Bolivian CF-negative strains, while P14 contained nine CS1+CS3+CS21-expressing clinical strains. Furthermore, environmental isolates expressing CS6+CS8 (E23 and E25) share the same PhP profile as four other clinical Bolivian strains expressing CS14, CFA/I, and CF-neg from 2009.

There was also a notable correlation between the phylogenetic tree of 362 ETEC (Fig. 1b) and the dendrogram of Bolivian isolates (Fig. 5). Our PhP data showed that strains P1 and P3, characterized as members of the ETEC lineage L19, clustered with isolates P2 (CS5+CS6) and P4 (CS14 and CF-neg) from ETEC lineages L5 and L17, respectively. P9 (CS23-expressing isolates), slightly more distant in the dendrogram, belonged to L16 and were linked to a group of primarily CF-negative isolates with unique PhP types and an uncharacterized lineage. According to von Mentzer’s study (18) and Fig. 1b, the closest lineages to L19 were L20 (CS14, CS6, and CF-neg), L5 (CS5+CS6), and L17 (CS6, CS14, and CF-neg), while L16, with diverse CF profiles, represented a more distant lineage. The L15 isolate 169 LAF was an exception, as the PhP method misplaced it with the L19 isolates. However, the L11 ETECs expressing CS23 found in water and clinical samples (E21 and 149 B7) fell into the P17 cluster, composed of distantly related L4, L6, and L9 lineages. Strains from these clusters were predominantly CFA/I+CS21 and isolated in Bolivia. In another example, Fig. 5 showed that strains P8 expressing CS23 (ET14, ET17, ET19) from lineage L15 clustered with other CF-negative Bolivian clinical strains with a different PhP type (P7) but the same lineage. The P14 and P19 strains, which included L1 and L4 isolates, were the closest to the L15 isolates. According to the tree in Fig. 1b, L1 is closely related to L15 (Fig. 5; Table S5).

In summary, the PhP method applied to this extensive ETEC collection revealed that some strains of this study shared similar metabolic fingerprints with previously collected clinical isolates in Bolivia and other endemic countries. The population structure of CS23 ETEC, based on the PhP method, exhibited high complexity with multiple PhP types spread throughout the dendrogram. Although only 69 isolates had their ETEC lineage identified, the PhP dendrogram resembled the population structure of the ETEC clades based on genomic data, suggesting stable combinations of chromosomes and plasmids with conserved metabolic fingerprints. Further inclusion of well-characterized ETEC strains is needed to enhance the discriminatory power of the PhP method.

### Antimicrobial resistance in ETEC strains during the past 12 years

Only ETEC strains previously isolated in Bolivia (104/152 strains) and those obtained in this study were included for the screening of phenotypic resistance to antibiotics against nine antibiotics using the AREB (Antibiotic Resistance in Environmental Bacteria) method (see Materials and Methods section). As described in Table 3, the total percentage of resistant, sensitive, and MDR strains, as well as the MAR index between isolates from this study and the retrospective collection of Bolivian strains, did not exhibit significant differences. However, a statistically significant increase in the MAR index was observed from 2007 to 2008 [analysis of variance (ANOVA), *P* = 0.0004; Fig. S4]. Although resistance to ciprofloxacin and nalidixic acid has risen since 2009, the number of resistant strains remains low. These findings indicate that while resistance to clinically relevant antibiotics has not increased substantially over the past decade in this set of strains, almost 50% of ETEC isolates displayed MDR.

**TABLE 3:** Comparison of annual resistance rates of Bolivian ETEC isolates^*a*^

Years	No. of ETEC analyzed	% of ETEC isolates												Average	
		Antibiotics									AMR patterns				
Collection of Bolivian strains		AMP	CTX	POD	CIP	NAL	GEN	TET	CHL	TMP	S	R	MDR	MAR	R
2002	1	100.0	0	0.0	0.0	0.0	0.0	100.0	0.0	100.0	0	100.0	0.0	0.20	2.40
2006	10	50.0	20	20.0	10.0	10.0	0.0	60.0	20.0	60.0	30	30.0	40.0	0.30	1.00
2007	19	36.8	0	0.0	0.0	5.3	5.3	21.1	10.5	21.1	57.9	31.6	10.5	0.11	2.68
2008	73	79.5	11	19.2	2.7	13.7	8.2	52.1	15.1	52.1	11	31.5	57.5	0.30	5.00
2009	1	100.0	100	100.0	0.0	0.0	100.0	0.0	0.0	0.0	0	0.0	100.0	0.56	2.62
Sub-total	104	69.2	10.6	16.3	2.9	11.5	7.7	47.1	14.4	47.1	21.2	31.7	47.1	0.32	2.7
*This study*															
2013–2014	30	53.3	0	0.0	10.0	43.3	0.0	33.3	20.0	46.7	20.0	46.7	36.7	0.22	2
Total	134	65.7	8.2	12.7	4.5	18.7	6.0	44.0	15.7	54.5	20.9	35.1	44.8	0.26	2.3

^*a*^ AMP, ampicillin; CTX, cefotaxime; POD, cefpodoxime; CIP, ciprofloxacin; NAL, nalidixic acid; GEN, gentamicin; TET, tetracycline; CHL, chloramphenicol; TMP, trimethoprim; S, sensitive; R, resistant; MDR, multidrug resistance; MAR, multiple antibiotic resistance.

## DISCUSSION

Monitoring the transmission of diarrheal pathogens and their antibiotic resistance patterns is crucial for evaluating outbreak risks and treatment strategies in countries with endemic diarrheal diseases. Factors such as inadequate clean water, poor sanitation (1), and limited surveillance and laboratory resources, that is, genomic sequencing, worsen disease and AMR burden (28). In Bolivia, studies have mainly focused on virulence and antibiotic resistance in clinical ETEC isolates (24, 29, 30), but increasing evidence points to frequent detection of ETEC in drinking and environmental water (11, 12, 14, 31, 32). By employing WGS and the PhenePlate screening method, we integrated two settings of the One Health concept to provide insight into the diversity and complexity of ETEC isolates from surface water and diarrheal cases. This approach allowed us to identify the transmission of ETEC clones positive LT+STh CS23 across environmental and clinical settings. Our findings suggest that the Choqueyapu River may serve as a reservoir for such bacteria and emphasize the inclusion of CS23 in future and retrospective epidemiological studies. The diverse virulence profiles of clinical ETEC compared to those from the Choqueyapu River indicate possible alternative reservoirs or transmission routes.

Our study has provided evidence of the spread of ST410 and ST218 LT+STh CS23-ETEC strains between hospitals and the environment, with identical CS23 alleles found in both stool samples and the Choqueyapu River. The genetic relationship of CS23 with porcine or plant-derived virulence factors F4 fimbria and other F4-like fimbriae (4) also raise the possibility that contaminated vegetables or meat could be potential vehicles for the transmission to the local population, as all environmental CS23-positive ETEC isolates were obtained from water samples near or on farmlands in the southern part of La Paz, where contaminated vegetables may be sold in over 80 markets in La Paz and El Alto. These findings underscore the considerable risks the Choqueyapu River poses to the local community’s health due to the pathogen transmission through water and food sources.

Regarding the detection and characterization of *E. coli* strains circulating between environmental and clinical settings, several studies found that environmental *E. coli* isolates possess different virulence genes traits and exhibit different antimicrobial resistance patterns as well as that they retain the capability to be cytotoxic in cell culture (13, 33, 34). ERIC (Enterobacterial Repetitive Intergenic Consensus)-PCR revealed a high degree of similarity among clinical and water isolates, suggesting the relevance of water as a potential route of transmission of enteric pathogens (33). Moreover, pulsed-field gel electrophoresis showed that some ETEC clones isolated from water sources were also detected in clinical samples (13).

ETEC is a significant etiological agent associated with childhood diarrhea in Bolivia (10). Previous molecular epidemiology studies, based on PCR detection of prevalent CFs (CFA/I, CS1-8, CS14, CS17, CS21) (24, 29, 35, 36), found that CFA/I, CS14, and CS17 are the most common CF among diarrhea-associated ETEC strains. Although 30%–40% of all ETEC strains remained negative for any CF, in the last 10 years, research on CF-negative strains has led to the discovery of new CFs such as CS23 (4). The integration of WGS provided a better resolution for the characterization of CFs and allowed the identification of non-traditional CFs from bacterial genomes, often neglected by surveillance and multicenter studies (17). WGS of 125 ETEC isolates from diarrheal cases in Chile showed that, in addition to the prevalence of CFs similar to previous global studies, the novel CF CS23 was present in 4.8% of the isolates, which indicates that CS23-expresssing ETEC circulates in the region (37). Our WGS-based CF identification characterized not only the CS23 operon in 19 ETEC isolates but also five novel variants of this colonization factor and four genomes (51 LAE, 250 B7 (1), 724 B9 (1), and ET6) harboring a gene cluster encoding potential chaperone-usher (CU)-assembled pili. Interestingly, high-risk ST410 isolates found in both clinical and environmental samples consistently contained the same CS23 allele (AalE3). Further functional studies are needed to verify the expression, assembly, and adherence patterns of the putative CS23 and CU-pili.

The population genomics of clinically relevant ETEC isolates from indigenous travelers and asymptomatic patients have been extensively studied, demonstrating remarkable genomic diversity (18, 38 – 40) and distinct lineages with conserved plasmid-encoded virulence profiles (18, 21). Our phenotypic and genomic approach similarly uncovered significant diversity among CS23 ETEC isolates in terms of biochemical fingerprints (PhP types), STs, and *E. coli* phylogroups (A, B1, C, and D). Comparing our data with 362 globally distributed clinical ETEC genomes from von Mentzer’s study, non-CS23 Bolivian isolates were found to belong to established, clonally distributed ETEC lineages (L1–L5) with a conserved combination of toxins, virulence factors and chromosomal background (18, 21). In contrast, our CS23-positive isolates span multiple lineages, with most clinical isolates being CF negative. Additional analysis of the longest contig (89,895 bp) containing the CS23 gene cluster suggests that the plasmid background is conserved, similar to other ETEC plasmids (21). Interestingly, a pattern is evident between the chromosomal background and the CS23 AalE variant, and the BLASTn analysis shows that the CS23 gene clusters of two isolates, MALC (1) and ET-26, are located on plasmids; however, further analyses are required to determine whether the plasmids harboring the CS23 gene cluster are conserved. These findings imply that the presence of CS23 in both environmental and clinical isolates may not indicate a clonal outbreak. Instead, the location of CS23-positive ETEC in the terminal branches of the phylogenetic tree of representative ETEC genomes suggests a recent acquisition (post-2011) of mobile genetic elements, that is*,* plasmids, which contributed to a faster diversification of these strains across different lineages. This phenomenon has been observed in *E. coli* genomes from animal and environmental sources (41).

Previous studies have shown that ETEC outbreaks can occur due to contaminated salad vegetables (42 – 44), with ETEC expressing CFs such as CS21 or virulence factors, such as flagella (45) and the *etpA* filaments (45), playing a role in adhesion to food-related surfaces. Our findings show that CS23-expressing ETEC strains were strong biofilm producers, which can contribute to their colonization and persistence (46) in various environments. The Choqueyapu River, contaminated with biofilm-inducing pollutants such as sub-MIC concentrations of antibiotics (47, 48), and its constant flow that provides oxygen and nutrients (49) creates the ideal conditions for growth and biofilm production. On the basis of these observations, we hypothesize that during dry seasons, bacteria accumulate in biofilms, then spread during the rainy season and re-enter the human host through contaminated water or food.

Ingested ETEC strains must adhere to intestinal cells to secrete toxins and initiate infection (1). Here, we demonstrate that clinical isolates exhibited greater adherence to CaCo-2 cells than environmental isolates, possibly due to the fact that clinical isolates were previously passed through a human host and were more apt to infect and adhere to epithelial intestinal cells than environmental strains. However, contrary to our results, Del Canto et al. (4) demonstrated that CS23 ETEC had a higher adhesion capacity than the CFA/I ETEC strain H10407. This discrepancy may be due to spontaneous mutations in the major fimbrial structural subunit AalE, which plays an essential role in the adherence to ETEC. The natural polymorphism identified in the AalE gene of our CS23 ETEC isolates could have negatively impacted bacterial adhesion, but research is needed to understand its role. Nevertheless, environmental isolates still possess virulence properties, as shown by our cytotoxicity assays.

The alarming levels of antibiotic residues present in the Choqueyapu River such as sulfamethoxazole (15) could contribute to the selection and spread of resistant ETEC in sediments and waters, potentially exacerbating the local and global AMR. On the other hand, ST410 is a high-risk MDR *E. coli* clone often associated with carbapenemase genes carrying *bla*_OXA-181_ and *bla*_NDM-5_ and with cross-sectorial transmission between wildlife, humans, companion animals, and the environment (50, 51). ST410 was previously found in soil samples from the Choqueyapu River and carried the *bla*_OXA-1_ gene (11). Studies have also found that *E. coli* ST410 is associated with vegetables on the municipal market in Ecuador (52) or surface water from Mexico’s agricultural drainage (53). The ST410 isolates from this study were not MDR but carried the CS23 operon combined with LT and STh. Another study assessing the MDR plasmid transfer from Choqueyapu’s waterborne bacterial communities to *E. coli* showed high-frequency transfer of the IncN plasmid carrying a wide range of ARGs and *intl1* (54). A concerning scenario would involve the combination of virulence genes and last-generation MDR genes within a single ST410 isolate. Since CS23-positive isolates were detected in children’s diarrheal stool and environmental water, such combinations could lead to the emergence of a diarrheagenic clone that spread more easily. The potential biofilm-forming ability of ST410 at GI (gastrointestinal) and environmental temperatures might further facilitate its survival and dissemination across different niches (55).

The high levels of MDR among ETEC isolates reported in this study agree with other reports in Bolivia (10, 29, 35), Latin America (56), other LMICs (57 – 59) as well as in other diarrheagenic *E. coli* (19). These findings mirror the high rates of empirical prescription, self-medication, and lack of regulation of antibiotics commonly observed in LMICs. Although low levels of quinolones were reported in this study, our retrospective evaluation and comparison with previous studies indicate an increasing trend over time of fluoroquinolone-resistant ETEC in Bolivia. This trend has also been observed among ETEC and EAEC isolates causing traveler’s diarrhea (60) and *Shigella* species associated with diarrhea (61). The impact of antibiotic resistance on ETEC-related diarrhea in children can be significant, particularly in settings where resistance is prevalent, leading to more severe or persistent diarrhea and complicating treatment options (62). Consequently, addressing AMR is a critical health issue that demands increased attention, funding, capacity building, research, and development (63).

The accurate discrimination of different bacterial isolates of the same species but different origins is crucial for effective infection prevention and control. We evaluated the performance of a fast and cost-effective phenotypic detection method compared to the genome-based MLST typing method. Our results indicate that the PhP assay is a reliable and useful tool for studying the bacterial population of coliforms in various sources, such as water (64 – 66) and intestinal flora (67, 68), as shown in previous studies. Additionally, our findings support the notion that the PhP assay provides results comparable to those of MLST-type resolution and reveals conserved metabolic fingerprints associated with specific *E. coli* sequence types (STs). This was demonstrated in our earlier studies of water pump station samples in Norway (64 – 66), and we have now extended this concept to the ETEC lineages, which showed conserved metabolic fingerprints. The use of the PhP assay, which can identify MDR pandemic clones ST131, ST648 (66), and the high-risk clone ST410, makes it a useful screening tool in resource-poor settings where sequencing multiple samples is unaffordable.

The association between ETEC expressing CS23 and diarrhea can only be speculated due to the low number of samples analyzed. Furthermore, a specific metabolic fingerprint could not be identified, as the level of diversity in the ETEC isolates expressing CS23 was high and the resolution of PhenePlate was low.

In conclusion, this study highlights the prevalence of CS23-positive ETEC isolates in environmental water in Bolivia and their circulation between this environment and human hosts. This finding suggests a broader distribution of CS23 than previously anticipated, advocating for its inclusion in ETEC epidemiology and virulence potential studies. We also establish a versatile framework for tracking bacterial pathogens using the cost-effective PhenePlate system, applicable across all components of One Health. Coupled with genomic technologies, this approach promises efficient monitoring and timely detection of emerging pathogens, particularly in regions with minimal surveillance and limited data.

## MATERIALS AND METHODS

### Isolation of strains

#### Environmental isolates

The study area comprises different locations along the Choqueyapu River. This river crosses the city of La Paz, Bolivia, and is highly polluted due to the discharge of domestic and industrial wastewater (12). Surface water samples were collected from five locations downstream of the Choqueyapu River. The sampling sites included five communities: Palomar, Mecapaca, Lipari, Aranjuez, and Carreras. They are characterized by agricultural areas that use Choqueyapu waters to irrigate vegetable crops. Water samples (300 mL) collected in sterilized bottles and stored at 4°C were processed on the same day of the collection. The water samples were filtrated through 0.45-µm pore-sized cellulose membrane filters (Millipore Sigma Aldrich, St. Louis, MO) with a vacuum/pressure pump system (Pall Life Sciences, Ann Arbor, MI). For enrichment, the filters were incubated overnight in EC Broth (Oxoid, Basingstoke, Hampshire, England) at 37°C. The enriched EC Broth cultures were seeded on MacConkey agar (Oxoid) and incubated overnight at 37°C. From each water sample, a total of 20 *E. coli*-like lactose-positive colonies were selected and subjected to PCR to detect ETEC toxins (LT, STh, and STp) as previously described (29). The reference ETEC strain H10407 was used as a positive PCR control. LT and/or ST-positive isolates were considered ETEC and cryopreserved (LB-glycerol, 85:15%, vol/vol) at −80°C for further experiments.

#### Clinical isolates

Identification of ETEC isolates from fecal samples collected from children under 5 years of age with acute diarrhea attending Los Andres Hospital (El Alto city) and Materno Infantil Hospital (La Paz city) was carried out as described by Gonzáles et al. (24). The clinical isolates were stored in LB agar with 12% glycerol at −80°C at the Institute of Molecular Biology and Biotechnology (IBMB) until further evaluations. The frozen strains were grown overnight in EC Broth and then placed on MacConkey agar plates. The isolates were re-evaluated for ETEC virulence factors by PCR using primers targeting ETEC toxins and CFs (24).

### DNA extraction, whole-genome sequencing, assembly, and annotation

Genomic DNA was extracted as previously described with standard methods (18) using the DNeasy blood and tissue extraction kit (Qiagen) and eluted in 200 μL of MilliQ water. DNA concentration was measured using Qubit, and 50 ng of DNA was used for library preparation. Sequencing libraries were prepared using the MGI FS library prep set according to the manufacturer’s instructions. The TapeStation D1000 kit (Agilent) was used to evaluate library quality. Circularized DNA of equimolarly pooled libraries was prepared using the MGI Easy Circularization kit (MGI Tech). The DNBseq 2 × 100 bp paired-end sequencing was performed using a DNBSEQ G400 instrument (MGI) according to the manufacturer’s instructions. Raw sequencing reads were assembled using the Velvet assembler.

Annotated assemblies were produced using the pipeline described in Ref. (69). For each sample, sequence reads were used to create multiple assemblies using VelvetOptimiser v2.2 and Velvet v1.2.10 5 (70). An assembly improvement step was applied to the assembly with the best N50, the contigs were scaffolded using SSPACE v2.0 (71), and the sequence gaps were filled using GapFiller v1.11 (72). Automated annotation was performed using PROKKA v1.5 (73) and a genus-specific database (“Escherichia”) from RefSeq (74). All the software developed by Pathogen Informatics at the WSI is freely available for download from GitHub (Pathogen Informatics, WSI, https://github.com/sanger-pathogens/vr-codebase, Bio-Assembly-Improvement: Improvement of genome assemblies by scaffolding and gap-filling, Pathogen Informatics, WSI, https://github.com/sanger-pathogens/assembly_improvement) under an open source license, GNU GPL 3. The pipeline improvement step is also available as a stand-alone Perl module from CPAN (http://search.cpan.org/~ajpage/).

### Genomic profiling: virulence, phylogroups, and multilocus sequence type

The genomic toxin and CF profiles were determined in two steps: (i) running Abricate using a custom database (https://github.com/avonm/etec_vir_abricate) and (ii) BLASTn of genomes against the custom database (https://github.com/avonm/ETEC_vir_db) using the in-house script that creates a BLAST file that can be loaded into the Artemis Comparison Tool (ACT) (https://www.sanger.ac.uk/tool/artemis-comparison-tool-act/) along with the reference database and the annotated genome of interest. The phylogroup of ETEC strains was determined using ClermonTyping (v20.03) (75). The multilocus sequence type (MLST) of Bolivian isolates was predicted using ARIBA (v2.14.6) (76) following the Achtman scheme and the database “Escherichia coli #1” with the reads as input data.

### Phylogenetic analysis

The core genome was defined using Roary (77) with the split-paralogs option switched off and using mafft as the aligner (command: roary -p 20 g 60000 -e -n -s -f output_dir *.gff). Single-nucleotide polymorphism (SNP) sites (78) were used to extract SNP sites. Furthermore, the number of constant sites for each nucleotide (ATGC) was also determined using SNP sites. The relationship between Bolivian ETEC isolates and in context with the 362 previously published ETEC isolates (18) was investigated (Fig. 1a and b). Estimated maximum-likelihood phylogenies were generated with IQTree (v1.6.10) using the GTR+F+I model and 1,000 ultra-fast bootstraps (-bb), and the constant sites from SNP sites were included for linear scaling of the branch lengths (-fconst) (command: iqtree -s aln_file -mem 4G -nt 4 -bb 1000 m GTR+F+I -fconst).

The phylogenetic trees, along with metadata, were visualized using R (v4.0.2, 2020-06-22, URL: https://www.R-project.org/), explicitly using the R packages GGTREE (79) and GGPLOT2 (v3.3.2, https://ggplot2.tidyverse.org/).

### Genomic analysis of CS23 gene clusters identified in Bolivian genomes

CS23-positive isolates were analyzed using BLASTn against a reference database of known CFs, including CS23, and visualized in ACT. The gene clusters encoding CS23 were then manually extracted and individual genes were used as input for an all-vs-all BLASTn together with the F4 and CS23 references (https://github.com/avonm/ETEC_vir_db/blob/main/ etec_vir_master.fasta). The structural major subunit AalE of all CS23-positive isolates was extracted and aligned using mafft (80). The aligned sequences were used as input for IQTree (model: WAG+F+G4) (http://www.iqtree.org/) to build a phylogenetic tree. In total, five variants of AalE were evident, which was corroborated by pairwise alignment in Jalview of each AalE variant.

### PhenePlate and AREB analysis

A rapid, semiautomated, and computerized typing method for *E. coli* was used (the *E. coli* system, PhP-RE) (PhPlate Microplate Techniques AB, Stockholm, Sweden; http://www.phplate.se). This system is based on measurements of the kinetics of 12 biochemical reactions, performed in 96-well microplates. ETEC isolates were obtained from this study, as well as from Bolivia, and worldwide ETEC collections were subjected to the PhP assay as described elsewhere (66, 81). Briefly, pure *E. coli* colonies were picked from the agar plates using sterile tooth sticks and inoculated into PhP-RE plates, previously filled with 150 µL of PhP suspension medium per well. Ten microliters of the bacterial suspensions were transferred to the wells of all other columns of the PhP-RE plate. Simultaneously, 20 µL of the initial bacterial suspension of the PhP-RE plate was transferred to the first column of AREB plates containing 10 antibiotics [column 2-11: ampicillin (32 µg), cefotaxime (2 µg), ceftazidime (16 µg), chloramphenicol (32 µg), ciprofloxacin (4 µg), gentamicin (16 µg), nalidixic acid (32 µg), cefpodoxime (3 µg), tetracycline (16 µg), and trimethoprim (16 µg)] and filled with 200 µL of Iso-Sensitest Broth (Oxoid), then 10 µL of suspensions were dispersed in each column (2 – 12) of AREB plates. The plates were incubated at 37°C for 18–24 hours, and images of each plate were produced using a desktop scanner (HP G4050). Using PhenePlate software (PhPlate Microplate Techniques AB), images of the PhP-RE plate were transformed into absorbance data to generate the biochemical fingerprints of all the isolates, which were compared pairwise to each other, and the similarity between each pair was calculated to generate a dendrogram. The software also compared the amount of bacterial growth in each well of the AREB plate against the control well for the same bacterial isolate (column 12) and printed out resistance rates as 0 (no growth, susceptible), 1 (weak response, the result was controlled by visual inspection), and 2 (similar amount of growth as in the control well, resistant). For the final analysis, a weak response was considered to indicate resistance. The total antibiotic resistance in a population was measured using the multiple antibiotic resistance index (MAR), calculated as the mean proportion of resistance for isolates. The maximum possible MAR value is 1.00, obtained when all isolates are resistant to all antibiotics tested (82, 83).

### Antimicrobial susceptibility test

The sensibility to antibiotics not included in AREB plates was determined using the disk diffusion method according to EUCAST (European Committee on Antimicrobial Susceptibility Testing) guidelines (https://www.eucast.org/clinical_breakpoints/). The following antibiotic disks were tested: azithromycin (15 µg), amikacin (30 µg), tobramycin (10 µg), streptomycin (10 µg), tigecycline (15 µg), and trimethoprim-sulfamethoxazole 1:19 (25 µg). *Escherichia coli* ATCC 25922 and *Staphylococcus aureus* ATCC 25923 were used as reference strains. Strains that exhibit resistance to one or more agents in the last three different antimicrobial categories are considered multidrug resistant (MDR).

### Biofilm formation

As previously described, bacterial strains were tested to assess *rdar* morphotype formation (84). The bacterial strains were grown on LB-no salt agar (LBns) overnight at 37°C and resuspended in phosphate-buffered saline (PBS) 0.001 pH 7.4 (Sigma). The bacterial suspension was adjusted to an optical density at 600 nm (OD_600nm_) of 3, and 5 µL was spot inoculated onto LBns supplemented with Congo red (40 µg/mL) and Coomassie brilliant blue (20 µg/mL). The colony morphology was observed after incubation at 37°C or 28°C for 48 hours; photographs were taken after incubation, and the diameter was measured.

### Cell culture assays and infection

Caco-2 cells (human colon adenocarcinoma cells) were obtained from the American Type Culture Collection and were grown in Dulbecco’s modified Eagle’s medium (DMEM, Sigma-Aldrich) supplemented with 10% (vol/vol) fetal bovine serum, 1% streptomycin-penicillin, and 1% non-essential amino acids. Cells were grown in T-25 cell culture flasks at 37°C with 5% CO_2_ in an atmosphere of 5% CO_2_/95% air with constant humidity. Confluent cell culture flasks were trypsinized and subcultured when necessary. For bacterial infection experiments, cells were seeded at a density of 4,000 cells/cm^2^ in 24-well tissue culture plates containing treated glass coverslips to grow adherent cells. Clinical and environmental ETEC bacterial isolates previously characterized were grown overnight in LB agar plates at 37°C 1 day before infection. The same day of infection, bacterial strains were resuspended in sterile PBS, adjusting to a desired OD. Cell culture wells containing growing Caco-2 cells were washed three times with sterile PBS. For infection experiments, DMEM medium without antibiotics was used with 10% heat-inactivated fetal bovine serum (30 minutes at 56°C). Caco-2 cells were infected with previously prepared bacterial suspensions at an MOI of 200:1 for an incubation time of 3 hours at 37°C and a 5% CO_2_ atmosphere. Each bacterial strain was tested in triplicate.

#### Adherence assay

Infected Caco-2 cells were washed three times with sterile PBS to remove non-adherent bacteria. The Caco-2 cells were then fixed with methanol and dyed with Giemsa stain. The stained-glass coverslips were removed from the culture wells and mounted on a microscope slide (three glass coverslips per strain). The adhesion of each bacterial strain was measured by counting the number of adherent bacteria in a total of 20 microscope fields (magnification 1,000×). The number of infected and non-infected Caco-2 cells in the same microscope fields was also registered for further analysis.

#### Cytotoxicity assay

The live/dead viability/cytotoxicity kit for mammalian cells (Molecular Probes, Eugene, OR) was used to assess the cytotoxicity of ETEC bacterial strains in Caco-2 cells. Briefly, after Caco-2 cell infection with bacterial strains (described above), cell culture wells were washed three times with sterile PBS, and cells were incubated with 2 µM calcein AM and 4 µM ethidium homodimer-1 for 30 minutes at room temperature. After incubation, stained glass coverslips were removed from the wells and mounted on a microscope slide in triplicate per strain. Microscope slides were observed under a fluorescence microscope (Leica DM LB2, Wetzlar, Germany), where viable cells showed a green fluorescent color and non-viable cells showed a red fluorescent nucleus. Viable and non-viable Caco-2 cells were counted and registered for further analysis.

## Data Availability

The complete sequences of ETEC were deposited in the National Center for Biotechnology Information database within the BioProject SRP416785.

## SUPPLEMENTAL MATERIAL

Tables S1 to S5 msystems.00141-23-s0001.xlsx Tables S1 to S5.

Fig. S1 msystems.00141-23-s0002.tif Maximum likelihood tree based on the amino acid sequence encoding the major subunit AalE in CS23 positive isolates. BLASTp comparison revealed five different variants of AalE (AalE-1 through 5). The ETEC lineage (based on the phylogenetic tree in Figure 1a and the predicted MLST and the phylogroup of each isolate) are included in the heatmaps. The source of the isolate is color coded at the tips and the scale bar represents the substitutions per variable site.

Fig. S2 msystems.00141-23-s0003.tif Gene organization of contigs carrying the class 1 integron-integrase gene intI1 and ARGs. An arrow denotes the gene area. The ARGs, the intI1 gene, and other features are colored.

Fig. S3 msystems.00141-23-s0004.tif Effect of temperature on the formation of morphotypes in clinical and environmental ETEC isolates at 20 {degree sign} C and 37 {degree sign} C for 48 h.

Fig. S4 msystems.00141-23-s0005.tif Comparison of the MAR index per year. Isolates are presented as individual values with each group's mean and standard error plotted. MAR indicates >0.2 (dotted line) that isolates likely originated from areas of high antibiotic use or high-risk source (83). Ordinary ANOVA was performed to compare the mean between groups. ***, *P* < 0.001.

ASM does not own the copyrights to Supplemental Material that may be linked to, or accessed through, an article. The authors have granted ASM a non-exclusive, world-wide license to publish the Supplemental Material files. Please contact the corresponding author directly for reuse.
