# Supplementary figures and images for: Circulation of enterotoxigenic Escherichia coli (ETEC) isolates expressing CS23 from the environment to clinical settings
Source: mSystems. 2023 Sep 8;8(5):e00141-23. doi: 10.1128/msystems.00141-23 (PMC10654058; doi:10.1128/msystems.00141-23)

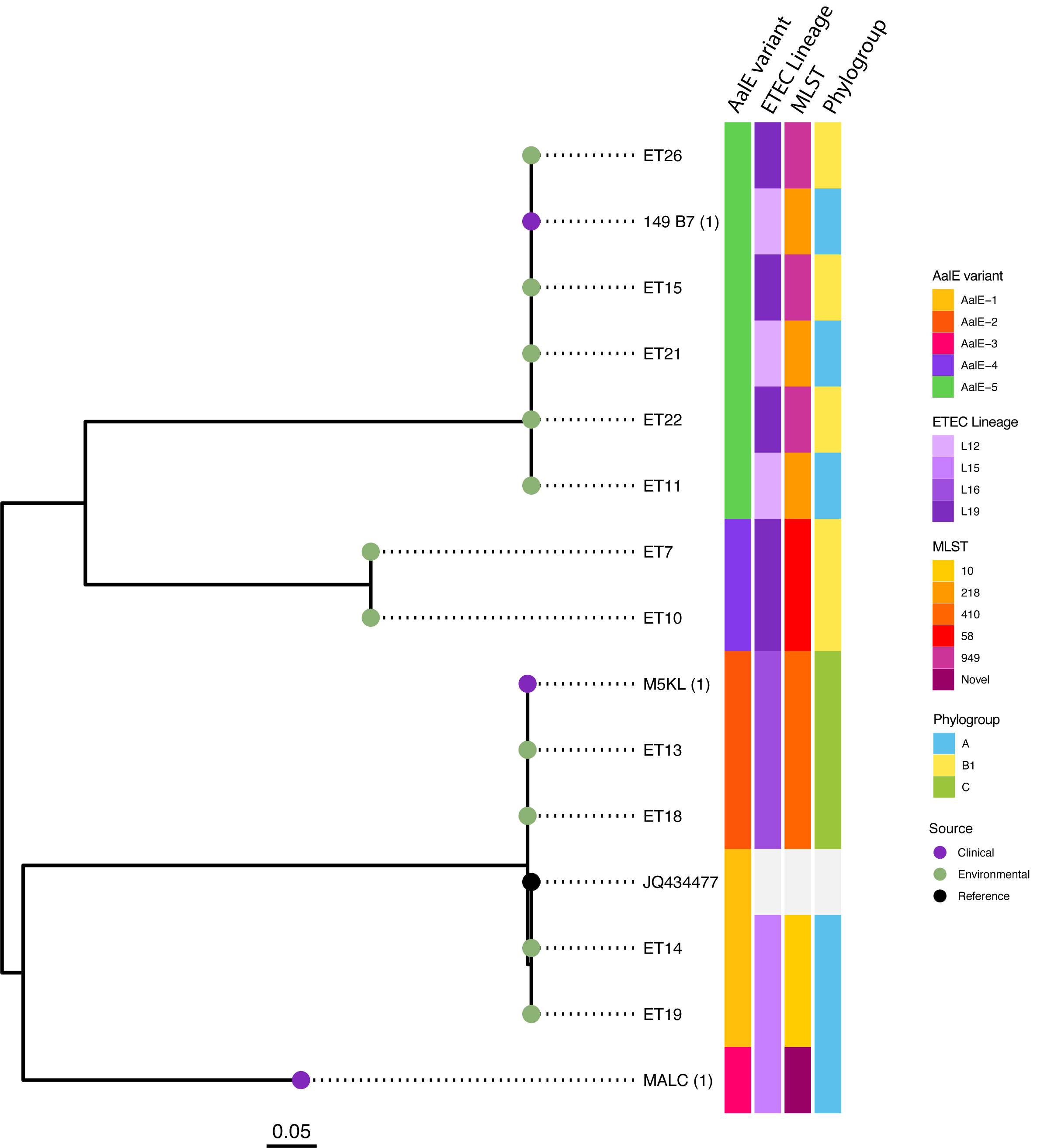

Supplement: Fig. S1 — Maximum likelihood tree based on the amino acid sequence encoding the major subunit AalE in CS23 positive isolates. BLASTp comparison revealed five different variants of AalE (AalE-1 through 5). The ETEC lineage (based on the phylogenetic tree in Figure 1a and the predicted MLST and the phylogroup of each isolate) are included in the heatmaps. The source of the isolate is color coded at the tips and the scale bar represents the substitutions per variable site. [file msystems.00141-23-s0002.tif]

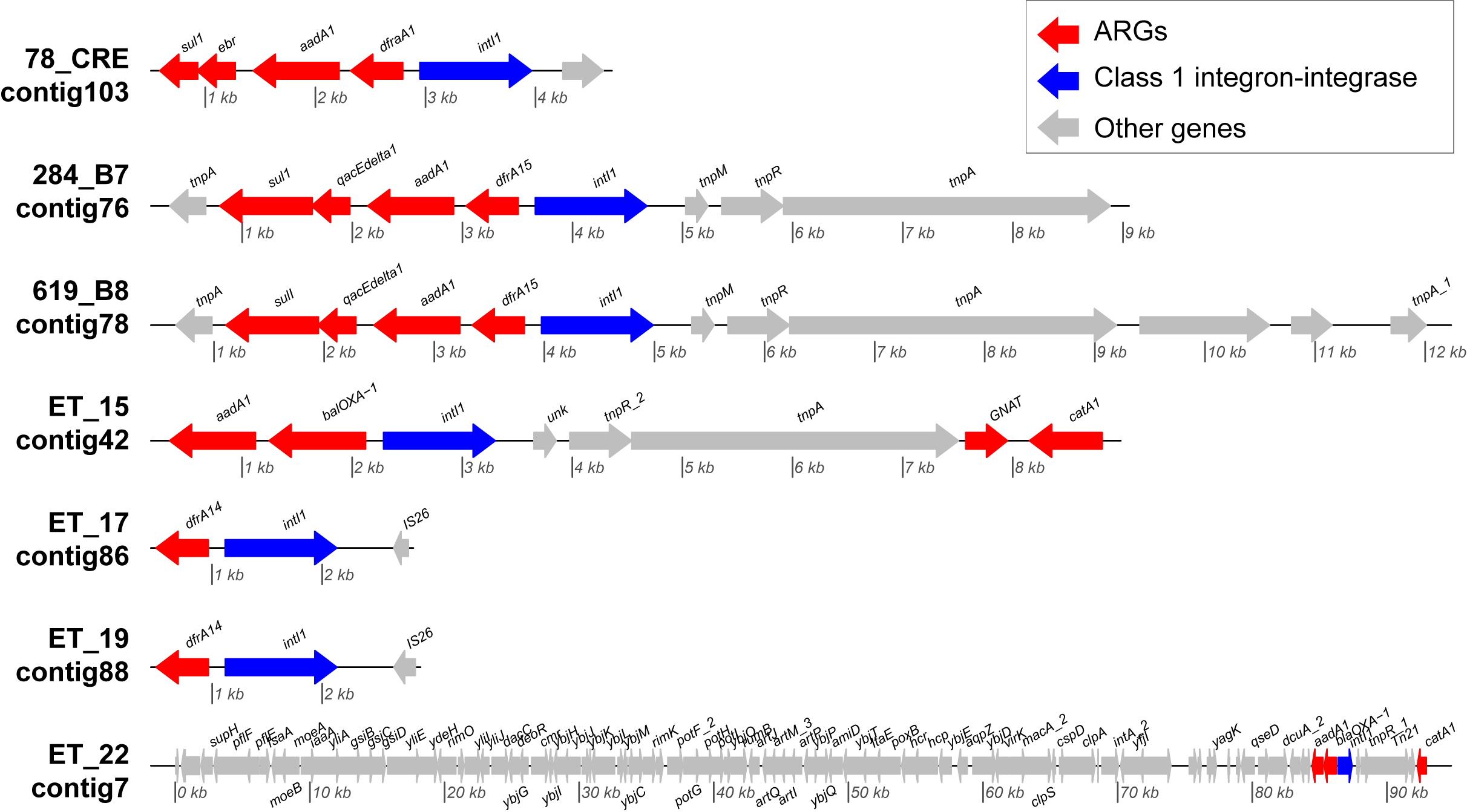

Supplement: Fig. S2 — Gene organization of contigs carrying the class 1 integron-integrase gene intI1 and ARGs. An arrow denotes the gene area. The ARGs, the intI1 gene, and other features are colored. [file msystems.00141-23-s0003.tif]

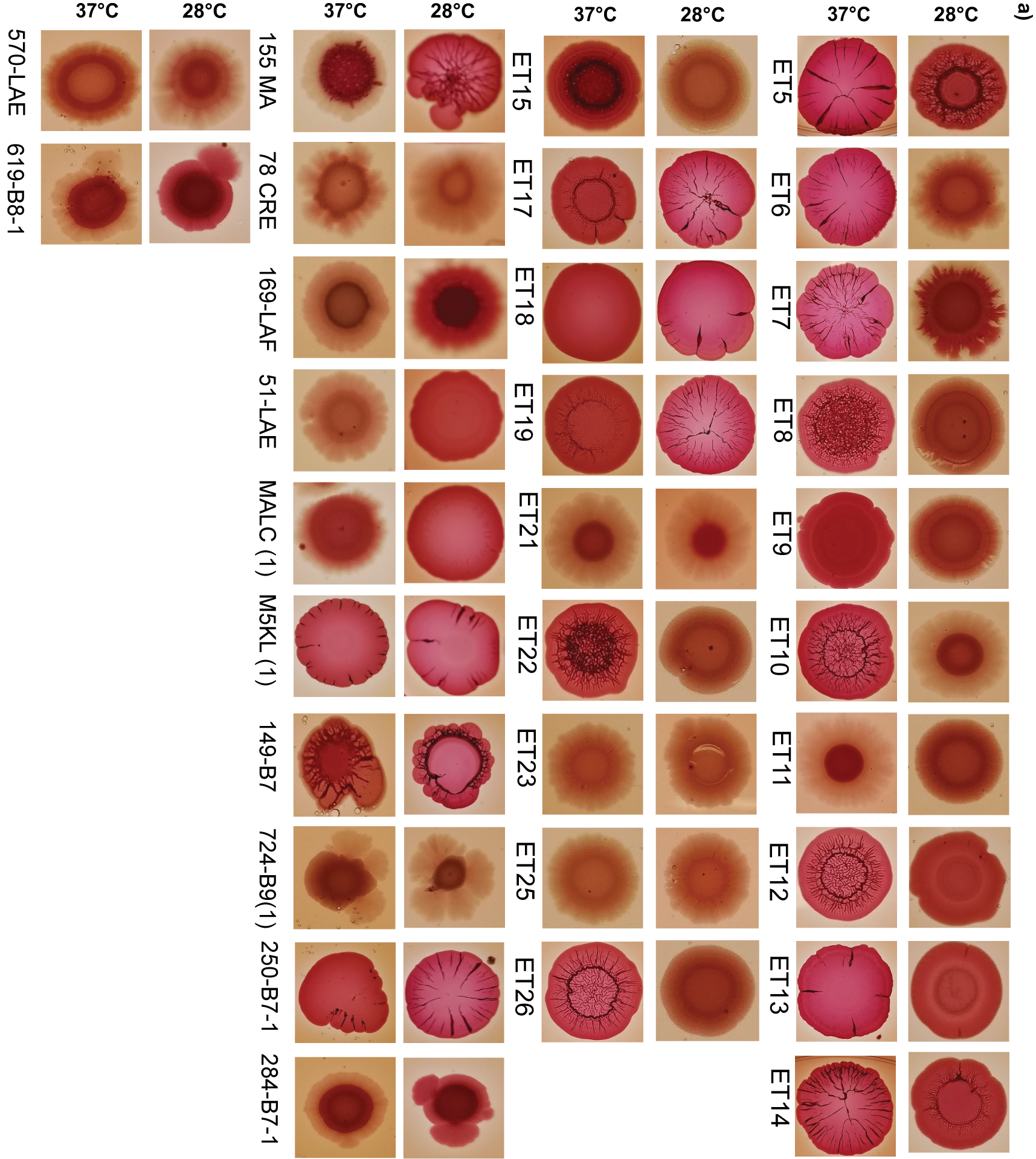

Supplement: Fig. S3 — Effect of temperature on the formation of morphotypes in clinical and environmental ETEC isolates at 20 {degree sign} C and 37 {degree sign} C for 48 h. [file msystems.00141-23-s0004.tif]

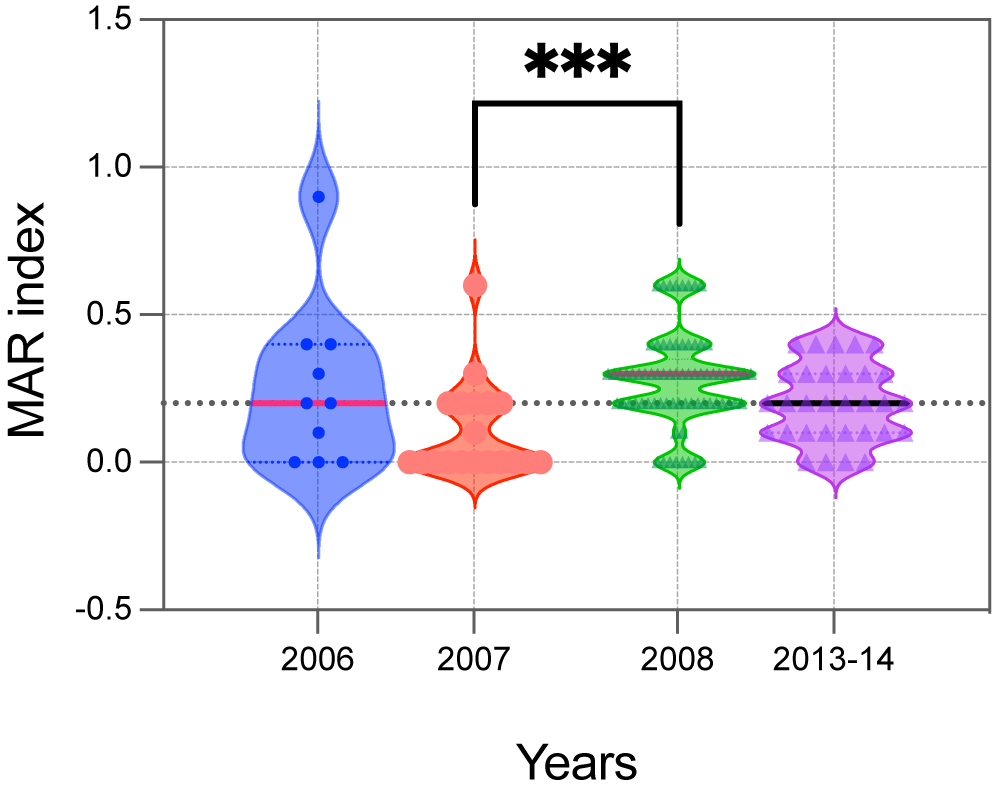

Supplement: Fig. S4 — Comparison of the MAR index per year. Isolates are presented as individual values with each group's mean and standard error plotted. MAR indicates >0.2 (dotted line) that isolates likely originated from areas of high antibiotic use or high-risk source (83). Ordinary ANOVA was performed to compare the mean between groups. ***, P < 0.001. [file msystems.00141-23-s0005.tif]
